# Association between asthma and IgG levels specific for rhinovirus and respiratory syncytial virus antigens in children and adults

**DOI:** 10.1016/j.jacig.2024.100342

**Published:** 2024-09-17

**Authors:** Marion Mauclin, Alicia Guillien, Katarzyna Niespodziana, Anne Boudier, Thomas Schlederer, Maja Bajic, Peter Errhalt, Kristina Borochova, Isabelle Pin, Frédéric Gormand, Raphaël Vernet, Jean Bousquet, Emmanuelle Bouzigon, Rudolf Valenta, Valérie Siroux

**Affiliations:** a Université Grenoble Alpes, INSERM U 1209, CNRS UMR 5309, Team of Environmental Epidemiology Applied to the Development and Respiratory Health, Institute for Advanced Biosciences, Grenoble, France; b the Division of Immunopathology, Department of Pathophysiology and Allergy Research, Center for Pathophysiology, Infectiology and Immunology, Medical University of Vienna, Vienna; c CHU Grenoble-Alpes, Grenoble, France; d the Department of Pneumology, University Hospital Krems and Karl Landsteiner University of Health Sciences, Krems; e the Pneumology Department, CHU de Lyon, Lyon; f Université Paris Cité, INSERM UMR 1124, Group of Genomic Epidemiology of Multifactorial Diseases, Paris; g Université Paris-Saclay, UVSQ, Université Paris-Sud, INSERM, Equipe d’Epidémiologie Respiratoire Intégrative, CESP, Villejuif; h Karl Landsteiner University, Krems

**Keywords:** Asthma, rhinovirus, respiratory syncytial virus, virus-specific IgG levels, epidemiology, children, adults

## Abstract

**Background:**

Viral infections in childhood, especially to rhinovirus (RV) and respiratory syncytial virus (RSV), are associated with asthma inception and exacerbation. However, little is known about the role of RV- and RSV-specific antibodies in childhood versus adult asthma.

**Objective:**

We sought to investigate associations between RV- and RSV-specific IgG levels and asthma phenotypes in children and adults.

**Methods:**

The analysis included 1771 samples from participants of the Epidemiological Study on the Genetics and Environment of Asthma (530 children; age [mean ± SD], 11.1 ± 2.8, and 1241 adults; age [mean ± SD], 43.4 ± 16.7, among whom 274 and 498 had ever asthma, respectively). RSV- and RV-specific IgG levels were determined using microarrayed virus-derived antigens and peptides. Cross-sectional associations between standardized RSV- and RV-specific IgG levels and asthma phenotypes were estimated by multiple regression models.

**Results:**

In children, ever asthma was associated with higher IgG levels specific to RV, especially to RV-A and RV-C, and to RSV (adjusted odds ratios [95% CI] for a 1 − SD increase in IgG levels were 1.52 [1.16-1.99], 1.42 [1.10-1.83], and 1.24 [0.99-1.54], respectively). These associations were stronger for moderate to severe asthma than for mild asthma. Conversely in adults, ever asthma was associated with lower RV-A, RV-B, and RV-C IgG levels (adjusted odds ratios [95% CI] were 0.86 [0.74-0.99], 0.83 [0.73-0.95], and 0.85 [0.73-0.99], respectively).

**Conclusions:**

Our results suggest that the association between respiratory virus–specific antibody levels and asthma varies during life, with asthma associated with higher levels of IgG to RSV, RV-A, and RV-C in children and lower levels of IgG responses to RV-A/B/C in adults.

Asthma is a disabling chronic disease characterized by respiratory symptoms (wheeze, breathlessness, chest tightness, and cough) and chronic inflammation of the airways. It is one of the most common chronic diseases in the world, affecting more than 300 million people,^1^ and it represents a major burden to individuals (particularly for those suffering from severe asthma) and society because of direct and indirect costs.^2^

Asthma is recognized as a complex multifactorial disease, resulting from genetic, environmental, and lifestyle factors. Respiratory infections are thought to be a major factor in the onset of the disease.^3^ It is well established that natural exposure as well as controlled inoculation with respiratory viruses, in particular respiratory syncytial virus (RSV) and rhinovirus (RV), induce symptoms of asthma and asthma exacerbations in patients susceptible to virus-induced asthma attacks.^4^^,^^5^ There is a large body of evidence demonstrating that early-life infection by RSV (eg, RSV bronchiolitis) is associated with an increased risk of childhood asthma.^6^ A large population-based study further showed that RSV infection in infancy increases the risk of childhood asthma.^7^ RVs, the most frequent viral pathogens found in respiratory tract infections, are the cause of the common cold. A large number of RV strains were characterized and classified into 3 genetic species—RV-A, RV-B, and RV-C—of which especially RV-A and RV-C seem to be important for asthma exacerbations.^8^, ^9^, ^10^ RV-induced wheeze in the first 4 years of life increases the risk of subsequent asthma, especially at age 7 years.^11^ This pattern of association of asthma with RV is considered to be even stronger than RSV-induced wheeze and persists into early adulthood.^12^

The detection of RV-specific antibodies with various microarrayed RV antigens and peptides from different RV species allowed a detailed and species-specific analysis of the RV-specific antibody responses.^13^^,^^14^ It has been shown that antibodies of RV-exposed individuals are mainly directed to the N-terminal region of the VP1 protein and are not neutralizing, and thus have been suggested to be responsible for recurrent RV infection.^10^^,^^15^ A study of an *in vivo* provocation test showed that the level of antibodies directed against the N-terminal region of VP1 increased in adults after experimental inoculation of RV16, and this increase was associated with more severe respiratory symptoms.^16^ Thus, the N-terminal VP1 epitope has been identified as a major target for the RV-specific antibody responses, and the specific IgG level to the N-terminal VP1 seems to be a good surrogate marker for virus exposure. Other studies have suggested higher RV-specific IgG levels among asthmatic children during wheeze or exacerbation.^17^^,^^18^ Thus, RV-specific antibody levels may be considered as serological surrogate markers for RV infections, and increases in RV-specific IgG levels after asthma exacerbations indicate RV infection as a trigger factor.^19^ However, most studies analyzing associations between RV-specific antibody levels and asthma have been performed in children, and thus data for adult asthmatic patients are rare.

The aim of this study was to analyze the association between RSV- and RV-specific IgG levels considered as surrogate markers for virus exposure, with “ever asthma” considered as the primary outcome, and asthma phenotypes in children and adults. In addition, we aimed to investigate the possible impact of allergic sensitization on the association between RSV- and RV-specific antibody levels and asthma.

## Methods

### Population and study setting

The study relies on the Epidemiological Study on the Genetics and Environment of Asthma (EGEA), a French multicenter cohort including participants with asthma recruited in chest clinics and their first-degree relatives as well as population-based participants recruited from electoral rolls for adults and from surgery departments for children. In total, 1443 adults and 604 children were recruited from 1991 to 1995 (EGEA1). Participants (348 asthmatic individuals and 416 individuals without asthma, both children and adults) were recruited in 5 French cities (Paris, Lyon, Marseille, Montpellier, and Grenoble). About 12 years later, enrolled individuals were invited for a follow-up visit (EGEA2), and 1543 participants accepted to participate and 58 new family members were included too. For both time points, individuals replied to a questionnaire and had a complete examination, including skin prick tests and blood samples. Written informed consent was obtained from all adult individuals and legal guardians of the children. Ethical approval was obtained for both surveys (Cochin Royal Hospital, Paris, for EGEA1 and Necker-Enfants Malades Hospital, Paris, for EGEA2).

Specific IgG responses to microarrayed RSV G protein^20^ and peptides derived from the N-terminal of VP1 of different RV strains^18^ were measured in EGEA1 (n = 531) and EGEA2 (n = 1360) serum samples, which were picked randomly and independently from the asthma status and respiratory symptoms.

### Asthma, asthma symptom score, and asthma severity definitions

Asthma-related outcomes were defined in EGEA1 and EGEA2 on the basis of validated questionnaires.^21^ Asthma was defined by a positive answer to the question “Have you ever had attacks of breathlessness at rest with wheezing?” or “Have you ever had asthma attacks?” or having being recruited as an asthmatic case in a chest clinic in EGEA1. The asthma symptom score ranged from 0 to 5 and was based on the number of respiratory symptoms in the last 12 months (wheezing with shortness of breath, woken up by a feeling of chest tightness, attack of shortness of breath at rest, attack of shortness of breath after exercise, woken up by an attack of shortness of breath, ever asthma, attack of asthma, and medication for asthma).^22^

Among the participants with ever asthma, asthma severity was defined according to the Global INitiative for Asthma 2002 guidelines, as previously used.^23^ “Moderate to severe asthma” was defined by at least 1 asthma attack in the last 12 months and any of the following: (1) use of inhaled corticosteroids (ICSs) in the last 12 months, (2) a clinical asthma score greater than 3, or (3) a clinical score of 2 and the use of asthma control therapy other than ICSs. Other individuals were classified as “mild asthma.” The clinical score (ranging from 0 to 7) was defined on the basis of asthma attack frequency (from 0 to 3), level of symptoms between asthma attacks (from 0 to 3), and hospitalization for asthma over the past 12 months (yes/no). The use of ICSs was considered in the last 12 months.

### Allergic sensitization

Allergic sensitization was defined by a positive result (mean wheal diameter of 3 mm greater than that of the negative control) to at least 1 of the 11 (EGEA1) or 12 (EGEA2) allergen extracts by skin prick testing (cat, *Dermatophagoides pteronyssinus*, *Cladosporium herbarum*, *Alternaria tenuis*, timothy grass, olive, birch, *Parietaria judaica*, ragweed, Aspergillus, and *Blattella germanica* in EGEA1 and the same plus cypress in EGEA2).

### RSV- and RV-specific IgG measurements and standardization

IgG levels to RSV G protein and to RV-derived N-terminal VP1 peptides from 18 RV-A strains, 9 RV-B strains, and 10 RV-C strains were determined in anonymized samples by microarray.^13^ The RV-derived N-terminal VP1 peptides are targets for nonneutralizing antibodies but represent major epitopes for the measurement of RV-specific antibody responses.^15^ The recombinant RSV G protein was expressed in *Escherichia coli* and purified by Ni-NTA affinity chromatography. IgG antibodies specific for the RSV G protein referred to as “RSV” were identified as serological markers for RSV infections.^20^ The specific raw IgG data from 21 assays were calibrated using control antigens to avoid assay effects, corrected for limit of detection by considering as missing values those below the background signal of the sample diluent, and selected for the analysis on the basis of the reproducibility of the assay.

For each subject, the sum of the IgG levels specific for the peptides from each RV species (ie, RV-A, RV-B, and RV-C) was calculated to obtain the cumulative peptide-specific IgG levels for the given RV species (see Fig E1, *A* and *B*, in this article’s Online Repository at www.jaci-global.org). Thus, cumulative RV-A–, RV-B–, and RV-C–specific antibody levels represent the sum of peptide-specific IgG levels from RV-A, RV-B, and RV-C, respectively. For RSV, IgG levels specific for the G protein were considered for each subject.

To obtain values of the summary variables with normal distribution, we applied the ordered quantile normalization transformation to each summary variable, using the “bestNormalize” R package from Ryan A. Peterson (Colorado School of Public Health) (see Fig E2, *A* and *B*, in this article’s Online Repository at www.jaci-global.org).

### Statistical analyses

For the descriptive analysis, percentages or medians with interquartile ranges of the RSV, RV-A, RV-B, and RV-C untransformed variables were used. We performed cross-sectional association analysis in EGEA1 (among children) and EGEA2 (among adults) between RSV- and RV-specific IgG variables (cumulative RV-A–, RV-B–, and RV-C–specific antibody levels) and asthma phenotypes (ever asthma, asthma severity, age of asthma onset, asthma symptom score, and asthma with use of ICSs) by applying logistic, multinomial, and negative binomial regression models, in agreement with the outcome considered. The familial dependence of the data was considered using a mixed model with a random intercept (R packages “lme4,” “lmerTest,” “mclogit,” and “MASS”). Regression models were adjusted for potential confounders including age (continuous), sex, body mass index (continuous), allergic sensitization, and season of blood sampling in EGEA1, and the same set of variables plus tobacco smoking in EGEA2. All the analyses were conducted separately in EGEA1 and EGEA2, and the results for children and adults were thus systematically interpreted separately. Multiple imputation method (m = 5) with R package “mice” was used to account for missing data on confounders (rate of missing data varying between 0% for age, sex, body mass index, and season and 5.3% for allergic sensitization).^24^

Further analyses were first stratified regarding allergic sensitization and then conducted by including an interaction term to assess a potential modifying effect of allergic sensitization in the association between respiratory virus IgG response and asthma outcomes (*P* < .1 for interaction considered significant). The analysis stratified on the allergic sensitization did not include a random intercept to account for the familial dependence because of convergence issue. We thus conducted a sensitivity analysis by including a single member per family to address the robustness of the results to the study design.

Interpretation of statistical tests was based on examining odds ratios (ORs) and their 95% CIs and precise *P* values (not whether *P* values were higher or lower than .05).^25^^,^^26^

## Results

### General characteristics of the population

**Fig 1:**
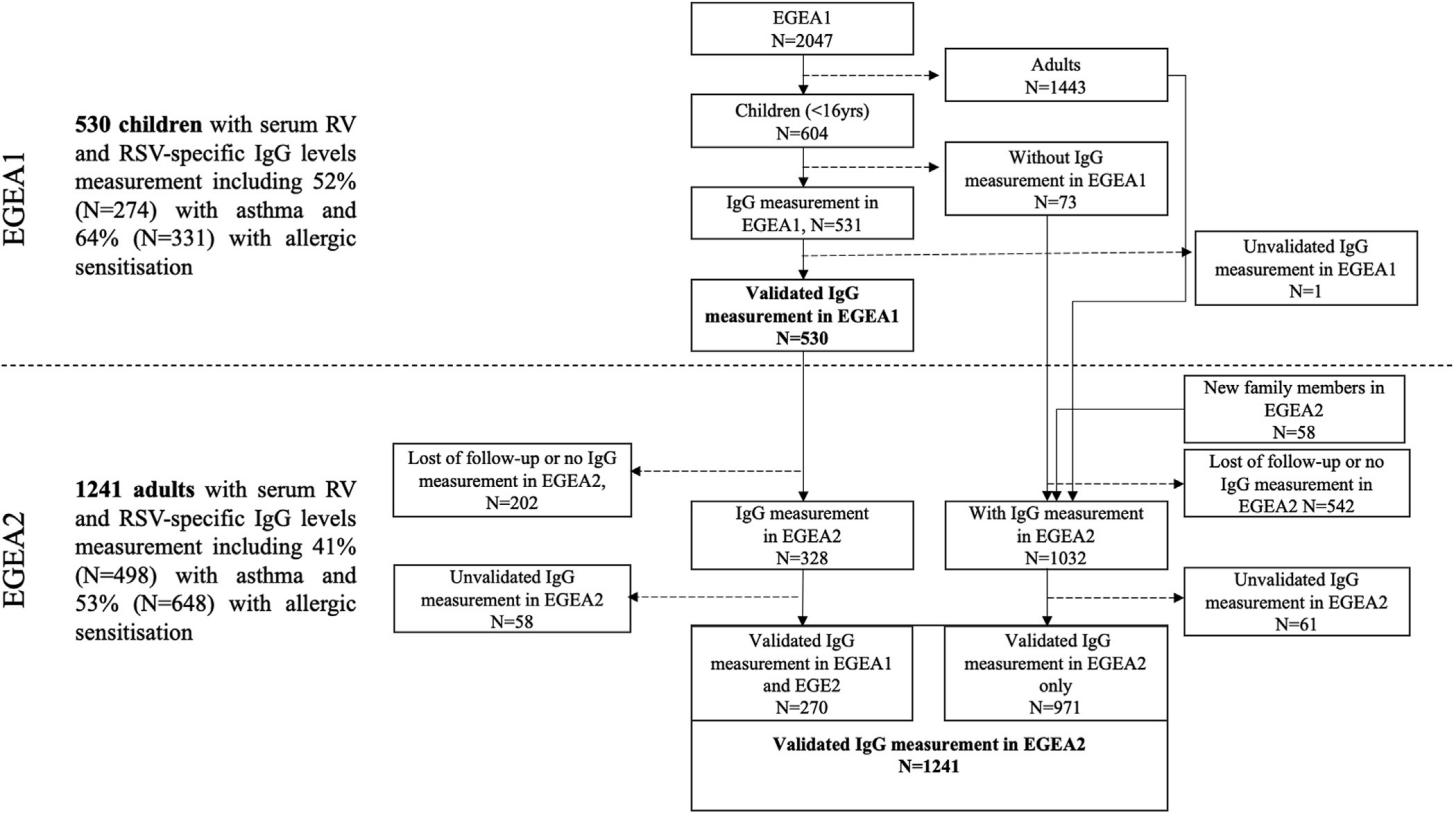
Design of the study. Flowchart of the study population indicating the number of subjects with valid IgG measures in EGEA1 and EGEA2 populations.

The study included 530 children in EGEA1 and 1241 adults in EGEA2 with valid measures of RSV- and RV-specific IgG responses, among whom 270 participants had been measured at the 2 time points (Fig 1). Among the children in EGEA1 (57% boys; mean age, 11 years), 52% ever had asthma and among those with asthma, 28% had moderate to severe asthma and 61% had an age of asthma onset before age 4 years (Table I). Sixty-four percent of children had allergic sensitization. Among the adults in EGEA2 (48% male; mean age, 43.5 years; 22% current smokers), 41% ever had asthma including 17% with moderate to severe asthma and 34% with adult-onset asthma (>16 years). Fifty-three percent of adults had allergic sensitization.

**Table I:** General characteristics of the study population

Characteristics	Children in EGEA1 (n = 530)	Adults in EGEA2 (n = 1241)
Sex: male	303 (57)	595 (48)
Age (y), mean ± SD	11.1 ± 2.8	43.5 ± 16.7
BMI (kg/cm^2^), mean ± SD	17.8 ± 2.9	24.4 ± 4.3
Exposure to passive smoking	208 (42)	—
Tobacco smoking		
Never smoker	—	630 (51)
Former smoker	—	339 (27)
Active smoker	—	270 (22)
No. of pack years		
0	—	609 (50)
0-10	—	366 (30)
10-20	—	113 (11)
>20	—	119 (9)
Season of data collection		
January-March	151 (29)	301 (24)
April-June	112 (21)	388 (31)
July-September	117 (22)	224 (18)
October-December	149 (28)	328 (26)
Socioprofessional category		
Executive	—	436 (36)
Technician	—	496 (41)
Worker-farmer	—	164 (14)
No profession	—	115 (9)
Allergic sensitization	331 (64)	648 (53)
Ever asthma	274 (52)	498 (41)
Severity of asthma		
No asthma	252 (51)	743 (63)
Mild asthma	170 (35)	365 (31)
Moderate to severe asthma	67 (14)	73 (6.2)
Age of onset of asthma		
No asthma	252 (49)	743 (62)
<4 y	161 (31)	138 (11)
4-16 y	103 (20)	167 (14)
>16 y		158 (13)
Asthma symptom score		
0	220 (45)	544 (46)
1	57 (12)	298 (25)
2	54 (11)	157 (13)
3	63 (13)	85 (7)
4	45 (9)	75 (6)
5	50 (10)	33 (3)
Asthma associated with ICS use in the last 12 months		
No asthma	252 (48)	743 (60)
Ever asthma without ICSs	228 (44)	292 (24)
Ever asthma with ICSs	42 (8)	204 (16)

Results are presented as n (%), unless otherwise mentioned.

*BMI*, Body mass index.

**Fig 2:**
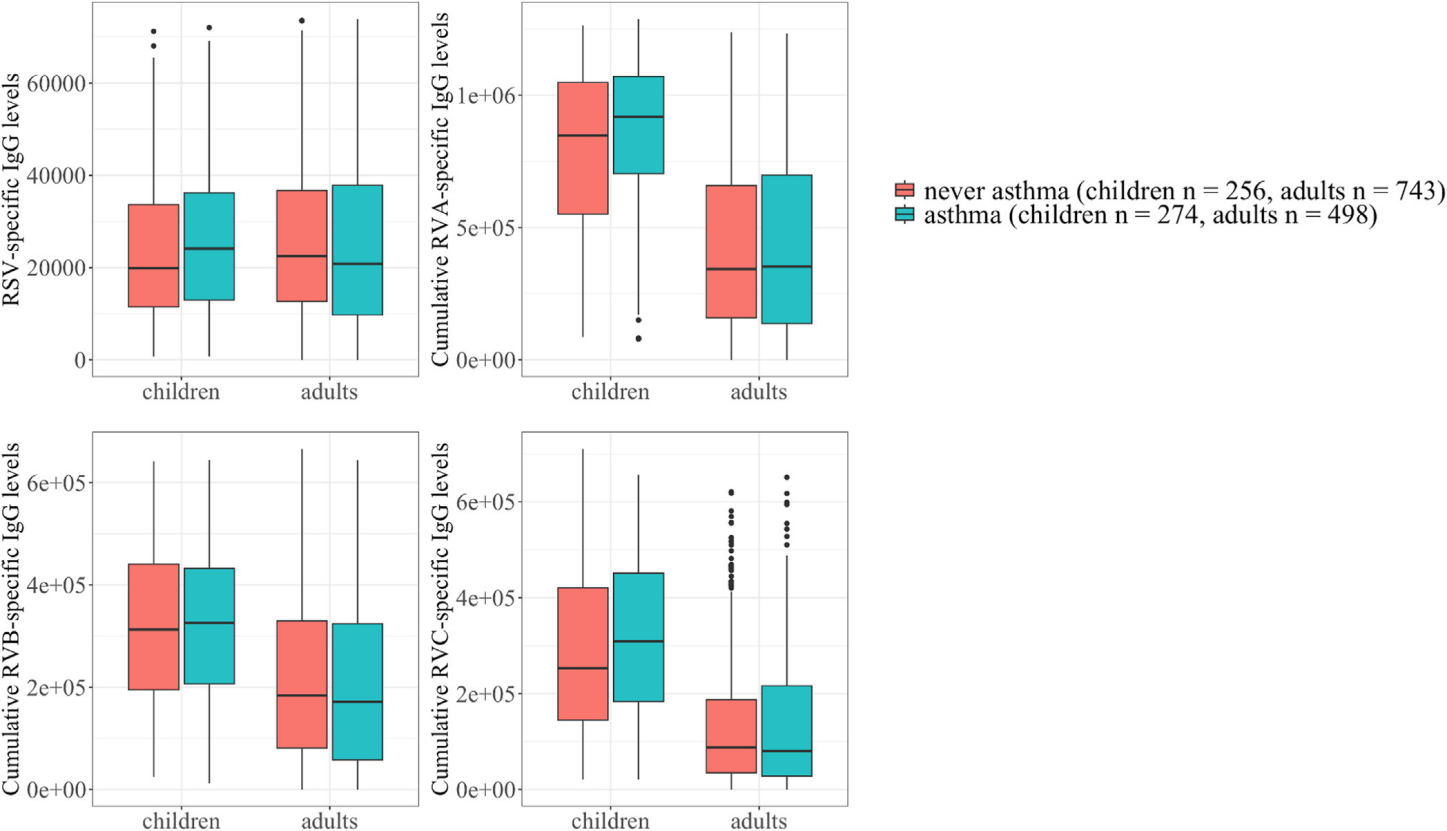
Antibody levels specific to RSV, RV-A, RV-B, and RV-C according to the ever asthma status (in children in EGEA1 and in adults in EGEA2). Shown are distributions of virus-specific IgG levels presented as median and interquartile ranges and expressed in fluorescence intensity values (y-axes). IgG data are demonstrated as boxplots for subjects without (*orange*) or with (*blue*) asthma in EGEA1 (children) and in EGEA2 (adults) subpopulations (x-axes).

The descriptive statistics (median and interquartile range) of the distribution of RSV and RV-A, RV-B, and RV-C IgG levels by the asthma status are presented in Table E1 (in the Online Repository available at www.jaci-global.org) and represented in Fig 2. In children, median (Q1-Q3) of RSV-specific and cumulative RV-A–, RV-B–, and RV-C–specific IgG levels (calibrated values given in fluorescence intensity) among asthmatic children were 24,139 (12,957-36,224), 918,493 (704,236-1,070,947), 325,754 (206,779-432,295), and 309,142 (183,815-451,179), respectively; among children without asthma, they were 19,889 (11,490-33,675), 848,379 (551,257-1,048,015), 312,658 (195,077-440,749), and 253,380 (145,071-420,565), respectively.

In adults, median (Q1-Q3) of RSV-specific and cumulative RV-A–, RV-B– and RV-C–specific IgG levels (calibrated values given in fluorescence intensity) among adults with asthma were 20,829 (9,734-37,865), 352,390 (137,448-698,541), 171,559 (58,015-324,169), and 80,496 (28,266-216,681), respectively; among adults without asthma, they were 22,475 (12,676-36,738), 343,269 (158,879-658,835), 183,902 (81,194-329,566), and 87,844 (34,679-187,602), respectively.

The highest correlations were observed between RV-A– and RV-C–specific IgG levels (coefficient correlation [*r*] = 0.72 in children and 0.81 in adults), and the lowest correlations were observed between RSV- and RV-specific IgG levels (*r* = 0.21-0.27 in children; *r* = 0.32-0.42 in adults).

### Association of RSV-specific and cumulative RV-A–, RV-B–, and RV-C–specific IgG levels with asthma phenotypes

In children, ever asthma was associated with higher cumulative IgG levels to RSV, RV-A, and RV-C (adjusted ORs [95% CI] for a 1 − SD increase in IgG levels were 1.24 [0.99-1.54], *P* = .06; 1.52 [1.16-1.99], *P* < .01; and 1.42 [1.10-2.83], *P* < .01, respectively) (Table II). Overall, for RSV, RV-A, and RV-C IgG levels, there was a general pattern with stronger magnitude of association for moderate to severe asthma than for mild asthma, and for stronger positive associations for asthma with ICSs as compared with asthma without ICSs. Higher RSV-specific and RV-A– and RV-C–specific IgG levels were associated with a higher asthma symptom score (adjusted ORs [95% CI] for a 1 − SD increase in IgG levels were 1.12 [1.00-1.25], *P* = .05; 1.14 [0.99-1.31], *P* = .06; and 1.14 [1.00-1.30], *P* = .04, respectively). The associations did not differ markedly according to age of asthma onset. No association was observed between any asthma phenotypes and levels of RV-B IgG antibodies in EGEA1.

**Table II:** Association study of RV-A, RV-B, RV-C, and RSV with asthma phenotypes

	Children in EGEA1		Adults in EGEA2	
Asthma phenotypes	(with asthma, n = 274 of 530)		(with asthma, n = 498 of 1241)	
	Adjusted OR (95% CI)	*P* value	Adjusted OR (95% CI)	*P* value
**Ever asthma**				
RSV	1.24 (0.99-1.54)	.06	1.01 (0.89-1.14)	.92
RV-A	1.52 (1.16-1.99)	2.69 × 10^−3^	0.86 (0.74-0.99)	.04
RV-B	1.01 (0.78-1.31)	.96	0.83 (0.73-0.95)	6.94 × 10^−3^
RV-C	1.42 (1.10-1.83)	6.88 × 10^−3^	0.85 (0.73-0.99)	.03
**Asthma severity**				
RSV				
No asthma	1.00		1.00	
Mild asthma	1.16 (0.90-1.50)	.24	1.03 (0.89-1.18)	.71
Moderate to severe asthma	1.36 (0.99-1.89)	.06	0.88 (0.69-1.13)	.31
RV-A				
No asthma	1.00		1.00	
Mild asthma	1.43 (1.05-1.95)	.02	0.86 (0.73-1.02)	.09
Moderate to severe asthma	1.52 (1.02-2.25)	.04	0.67 (0.50-0.91)	8.7 × 10^−3^
RV-B				
No asthma	1.00		1.00	
Mild asthma	0.98 (0.73-1.32)	.89	0.84 (0.73-0.98)	.03
Moderate to severe asthma	0.89 (0.60-1.31)	.55	0.65 (0.50-0.85)	1.71 × 10^−3^
RV-C				
No asthma	1.00		1.00	
Mild asthma	1.26 (0.94-1.69)	.12	0.87 (0.73-1.03)	.10
Moderate to severe asthma	1.70 (1.17-2.47)	4.99 × 10^−3^	0.72 (0.54-0.96)	.03
**Age of asthma onset**				
RSV				
No asthma	1.00		1.00	
≤4 y	1.28 (0.99-1.66)	.06	1.14 (0.94-1.40)	.19
4-16 y	1.16 (0.87-1.54)	.31	1.03 (0.86-1.23)	.76
>16 y			0.90 (0.75-1.07)	.23
RV-A				
No asthma	1.00		1.00	
≤4 y	1.49 (1.08-2.06)	.02	0.85 (0.67-1.07)	.17
4-16 y	1.56 (1.11-2.21)	.01	0.91 (0.74-1.13)	.39
>16 y			0.77 (0.61-0.96)	.02
RV-B				
No asthma	1.00		1.00	
≤4 y	1.21 (0.88-1.65)	.24	0.89 (0.72-1.10)	.28
4-16 y	0.77 (0.55-1.08)	.13	0.86 (0.71-1.04)	.12
>16 y			0.75 (0.62-0.92)	4.39 × 10^−3^
RV-C				
No asthma	1.00		1.00	
≤4 y	1.31 (0.97-1.78)	.08	0.82 (0.65-1.04)	.10
4-16 y	1.58 (1.14-2.18)	5.44 × 10^−3^	0.94 (0.76-1.17)	.61
>16 y			0.75 (0.60-0.94)	.01
**Asthma symptom score**∗				
RSV	1.12 (1.00-1.25)	.05	1.01 (0.95-1.08)	.71
RV-A	1.14 (0.99-1.31)	.06	0.94 (0.87-1.02)	.13
RV-B	0.93 (0.81-1.06)	.30	0.93 (0.87-0.99)	.04
RV-C	1.14 (1.00-1.30)	.04	0.97 (0.90-1.05)	.42
**Asthma associated with ICS use in the last 12 mo**				
RSV				
No asthma	1.00		1.00	
Ever asthma without ICSs	1.05 (0.7-1.58)	.81	1.01 (0.86-1.18)	.93
Ever asthma with ICSs	1.41 (1.1-1.8)	6.68 × 10^−3^	1.01 (0.85-1.19)	.92
RV-A				
No asthma	1.00		1.00	
Ever asthma without ICSs	1.45 (0.91-2.3)	.12	0.95 (0.79-1.14)	.57
Ever asthma with ICSs	1.66 (1.24-2.23)	6.5 × 10^−3^	0.75 (0.61-0.91)	4.74 × 10^−3^
RV-B				
No asthma	1.00		1.00	
Ever asthma without ICSs	0.95 (0.6-1.52)	.84	0.85 (0.72-1)	.05
Ever asthma with ICSs	1.04 (0.78-1.39)	.79	0.83 (0.69-0.99)	.04
RV-C				
No asthma	1.00		1.00	
Ever asthma without ICSs	1.27 (0.81-1.99)	.3	0.94 (0.78-1.13)	.49
Ever asthma with ICSs	1.55 (1.17-2.05)	2.2 × 10^−3^	0.74 (0.61-0.91)	4.33 × 10^−3^

Multivariate associations between asthma-related outcomes and RSV- and RV-specific IgG levels in children and adults. Models, accounting for random effect on family, were adjusted in EGEA1 on age, sex, body mass index, season of blood sample, and allergic sensitization and further adjusted in EGEA2 on tobacco active smoking.

∗ Estimates for asthma symptom score are mean score ratios, which model the ratio of the mean asthma symptom score for each 1-unit increase in virus IgG response.

In adults, ever asthma was associated with lower levels of cumulative IgG levels to RV-A, RV-B, and RV-C (adjusted ORs [95% CI] were 0.86 [0.74-0.99], *P* = .04; 0.83 [0.73-0.95], *P* < .01;and 0.85 [0.73-0.99], *P* = .03, respectively) (Table II). For cumulative RV-A–, RV-B–, and RV-C–specific IgG levels, negative associations were slightly stronger for moderate to severe asthma than for mild asthma, for adult-onset asthma than for childhood-onset asthma, and for asthma with ICSs as compared with asthma without ICSs. Higher cumulative RV-B–specific IgG levels were associated with a lower asthma symptom score. No association was observed between any asthma phenotype and RSV IgG levels in adult asthmatic participants in EGEA2.

### Modifying effect of allergic sensitization on cumulative RV- and RSV-specific IgG levels with asthma phenotypes

**Fig 3:**
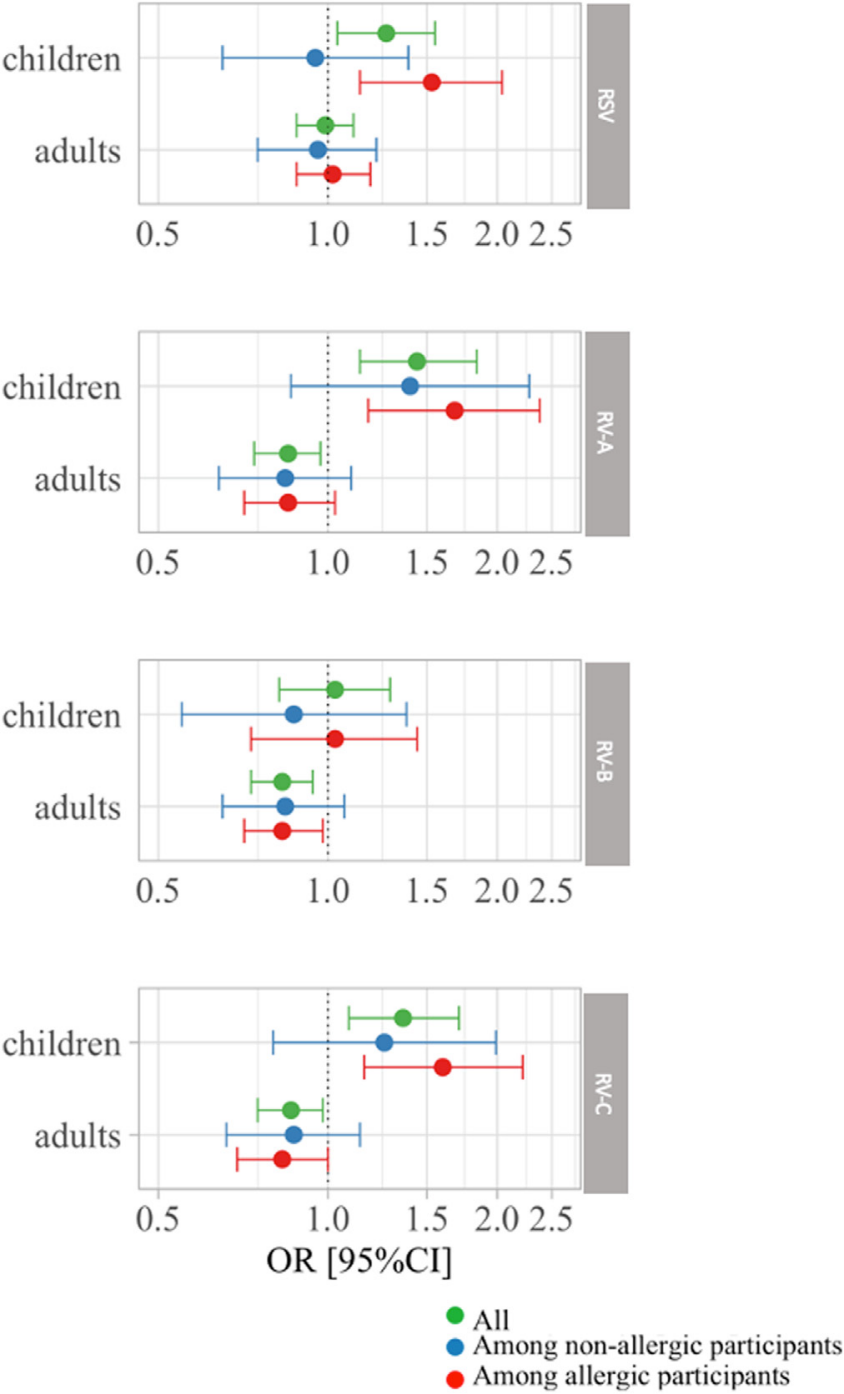
Association study of ever asthma with RSV- and RV-specific IgG levels in the whole population and after stratification according to allergic sensitization (in children in EGEA1 and in adults in EGEA2). Multivariate associations between ever asthma and RSV- and RV-specific IgG levels in children and adults, in all individuals, and in subgroups of allergic sensitized and nonsensitized individuals. Models, accounting for random effect on family, were adjusted in EGEA1 on age, sex, body mass index, and season of blood sample and further adjusted in EGEA2 on tobacco active smoking. For the model in the whole population, allergic sensitization was added as a cofactor.

In children, a statistical interaction was observed between RSV-specific IgG levels and allergic sensitization, with a stronger positive association between ever asthma and RSV-specific IgG levels among allergic sensitized children as compared with nonallergic children (*P* for interaction = .08) (Fig 3; see also Table E2 in this article’s Online Repository at www.jaci-global.org). Similar results were found for mild asthma, asthma with age at onset between 4 and 16 years, and asthma symptom score (Table E2). For the cumulated RV-specific IgG levels, results do not indicate statistical interaction with allergic sensitization on the asthma risk.

In adults, similar patterns of associations were observed in allergic and nonallergic adults, regardless of the virus-specific IgG levels and the asthma phenotypes considered. The sensitivity analysis including a single family member (321 children and 555 adults) showed similar results (see Table E3 in this article’s Online Repository at www.jaci-global.org).

## Discussion

To our knowledge, this is the first study to assess the association between specific immune levels to RV (species A, B, and C) and RSV and asthma phenotypes in a large population including both children and adults. In children, higher RSV and peptides-cumulative RV-A– and RV-C–specific IgG levels were associated with a higher risk of ever asthma, more specifically with moderate to severe asthma and ICS-treated asthma, and with increased asthma symptom scores. This pattern of association was stronger among children with allergic sensitization for RSV-specific IgG levels. On the contrary, in adults, higher peptides-cumulative RV-A–, RV-B–, and RV-C–specific IgG levels were associated with a lower risk of asthma, with slightly stronger association observed for moderate to severe asthma, adult-onset asthma, and ICS-treated asthma.

Our findings for children are supported by few previous studies. Two studies with limited sample size (n < 70) found a positive association between quantitative RSV IgG levels and ever asthma and severe asthma.^27^^,^^28^ A recent large population-based study (n = 1741) showed that RSV infection during infancy plays a critical role in the development of asthma in the first 5 years; not being infected with RSV in infancy was associated with 26% lower risk of asthma in the first 5 years as compared with infected children.^7^ Three other studies, also based on cohorts with moderate sample size (n < 250), found a positive longitudinal association between RV-A and RV-C IgG antibody levels and asthma, asthma-related episodes, severe asthma, and atopy.^14^^,^^18^^,^^29^

We found a stronger pattern of association between specific RSV immune levels and asthma among children with allergic sensitization, which is consistent with previous studies indicating a synergistic effect between virus-induced wheeze and atopy.^30^ Several *in vitro* studies have also linked the T_H_2 environment induced by allergy to impaired antiviral responses, especially to RSV infections, mediated by a deficit in type I and III interferon.^31^ Furthermore, studies suggest that virus-induced asthma exacerbations may be associated with an impaired innate immune response to RV, especially by interferon I and III pathways, thymic stromal lymphopoietin, and the IL-33 proteins.^32^^,^^33^ This may increase susceptibility to more severe viral infections. However, a recent study found opposite results, with an increased risk of nonallergic asthma at 5 years among children with a symptomatic RSV infection during infancy, whereas there was no association with allergic asthma.^7^ This may be explained by the fact that RSV can lead to asthma development through different pathways, both mediated by allergy and atopy or by other mechanisms, in particular in an inappropriate neural control of airways smooth muscle through β-adrenergic receptors, regardless of atopic status.^34^ A possible synergistic effect between RV infection and atopy in asthma is supported by *in vitro* studies showing that RV infections damage the respiratory epithelial barrier in cultured cells, which leads to increased allergen transmigration.^35^

Our results indicating stronger association between asthma and RSV-specific levels in children with early-onset asthma are consistent with the results of several studies that have identified the 17q21 genetic locus to be associated with a very specific subtype of asthma and early onset (<4 years)^36^ and induced by viral wheezing and maternal smoke during pregnancy.^37^^,^^38^ Furthermore, the positive associations observed for RV-A and RV-C but not for RV-B are in agreement with previous studies showing a wheezing effect stronger for RV-A and RV-C as compared with RV-B and support a differential pathogenicity between the RV strains.^39^^,^^40^

Asthma status by itself could induce a predisposition to develop enhanced viral infections. It is known that epithelial cells and innate lymphoid cells in airways of patients with asthma have an enhanced production of IL-13, IL-25, and IL-33 and a deficit in type I and III interferon responses that are needed for effective antiviral clearance.^41^^,^^42^ Therefore, a possible explanation could be that marked allergic airway inflammation, a marked characteristic in asthma, inhibits mucosal immune responses to viral infections, leading to increased viral replication and viral antigen load, which in turn enhance antibody responses.^43^^,^^44^

Our results in adults, showing a negative association between RV-A–, RV-B–, and RV-C–specific antibody levels and asthma, were *a priori* unexpected and indicate that the association between respiratory virus–specific antibody levels and asthma varies over the course of life. This pattern of association was not observed for RSV, and results for RV were robust to sensitivity analyses and coherent across the different asthma outcomes. To our knowledge, only 1 study based on 28 volunteers investigated the association between RV-specific immune levels and asthma in adults and found that 6 weeks after infection to RV16, the serologic increase was higher among those with moderate to severe asthma than among those without asthma.^16^ Contrary to our results, the VP1 RV-A (RV16) IgG levels at baseline were higher in asthmatic individuals, although this difference was not statistically significant. One can hypothesize that adults have acquired a protection against a broad range of RVs through previous infections, and are thus less likely to be reinfected and develop less antibodies with time. This hypothesis could partly explain differences observed between children and adults, as has been shown in a recently published article.^45^ However, this observation does not explain the inverse association between specific IgG levels and asthma observed between children and adults.

Our analysis indicates stronger positive associations in children and stronger negative associations in adults for ICS-treated asthma as compared with asthma not treated by ICSs. ICS use might be considered as a surrogate marker of asthma severity; therefore, these results reinforce the association observed with asthma severity. However, further studies are needed to investigate whether asthma treatments, and in particular ICSs, could affect immune responses to respiratory viral infections, and in particular specific IgG levels to RSV and RV.

One of the strengths of our study relies on the quantitative IgG assessment with a reproducible microarray technology that has been validated in longitudinal studies measuring alterations in virus-specific IgG by ELISA and microarray,^13^^,^^46^ allowing us to measure many peptides of interest of the 3 RV species and RSV, on a large cohort including a high proportion (about 50%) of participants with asthma. Moreover, the originality of this study is to include both children and adults. It thus allowed us to investigate a large range of asthma phenotypes, including childhood and adult asthma with early-onset asthma and late-onset asthma. The design of our study, with the measurement of antibody levels to respiratory viruses, is useful to evaluate the whole exposition to RV and RSV and not restrict the population to symptomatic or severe infections only. Our study also has some limitations. First, this study is a cross-sectional association study without any information on the last respiratory viral infection or the number of infections, and thus the temporality of the associations cannot be inferred. It is also difficult to assess the direction of the associations because a reverse causation cannot be ruled out. Note that the EGEA population, enriched with individuals with asthma by the study design, is not representative of the general population and therefore our results may not be generalizable. Furthermore, serologies reflect both viral exposure and host response, and it is possible that exposure to viral infection differs between individuals, particularly between children and adults, although this is unlikely for these common respiratory viruses with high transmission rates. We acknowledge that age of onset of asthma may be subject to recall bias, especially among adults with childhood-onset asthma. However, previous studies showed high accuracy of the self-reported age of asthma onset.^47^^,^^48^ Eventually, as in every epidemiological study focusing on a multifactorial disease, bias from other confounding factors may remain although regression models were adjusted on the principal confounders described in the literature.^45^

### Conclusion

Our study suggests that increased levels of IgG levels to RSV, RV-A, and RV-C species are associated with a higher risk for asthma in children, especially with moderate to severe asthma. The association between RSV-specific IgG levels and asthma was stronger among children with allergic sensitization. On the contrary, in adults, increased levels of specific IgG to RV-A/B/C were associated with a lower risk of asthma. These results showing different patterns of association across the course of life suggest that the different immune pathways involved in asthma follow one another over time, although further longitudinal studies are warranted to replicate these findings and specify the direction of these relationships.

**Key messages**

• Higher IgG levels to RSV, RV-A, and RV-C were associated with a higher risk of asthma in children.
• On the contrary, in adults, higher levels of RV-specific IgG levels were associated with a lower risk of asthma.
• High levels of RV-specific antibodies in asthmatic children may reflect multiple infections, whereas in asthmatic adults low levels may be indicative of extended protection against RVs.

## Disclosure statement

This study was supported in part by NIRVANA (grant no. ANR-19-CE36-0005) and by the Danube Allergy Research Cluster Program (DARC-ARC 2.0) of the country of Lower Austria.

Disclosure of potential conflict of interest: R. Valenta has received research grants from Worg Pharmaceuticals, HVD Biotech, and Viravaxx; and serves as a consultant for Viravaxx and Worg. The rest of the authors declare that they have no relevant conflicts of interest.

## Figures and Tables

**Supplementary Figure E1A:**
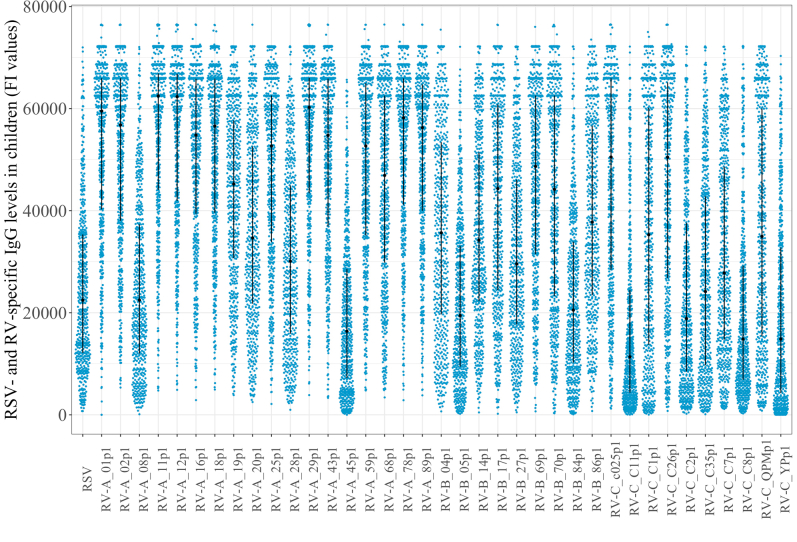
**Beeswarm plots of RSV- and RV-specific antibody levels, in children (part A, EGEA1, n=530), and in adults (part B, EGEA2, n=1241).** Distributions of virus-specific IgG data presented as median and interquartile range of the calibrated and corrected for the background level values (before normalization) and expressed in FI.

**Supplementary Figure E1B:**
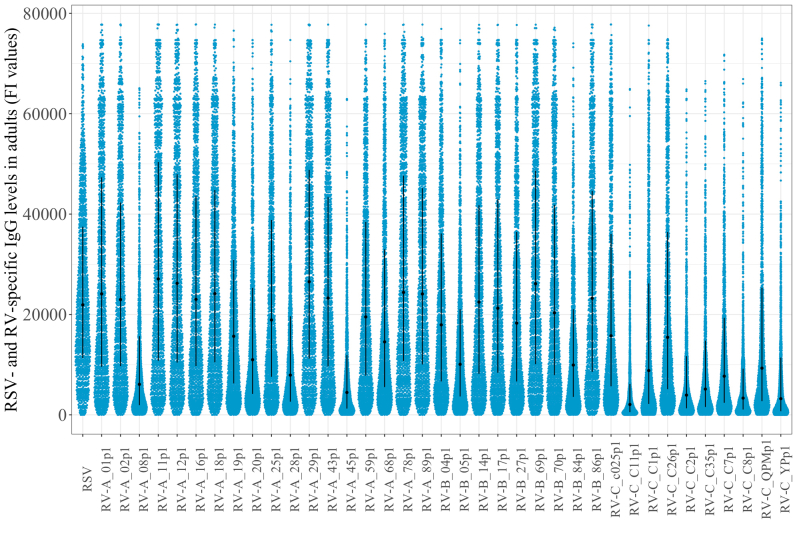
**Beeswarm plots of RSV- and RV-specific antibody levels, in children (part A, EGEA1, n=530), and in adults (part B, EGEA2, n=1241).** Distributions of virus-specific IgG data presented as median and interquartile range of the calibrated and corrected for the background level values (before normalization) and expressed in FI.

**Supplementary Figure E2A:**
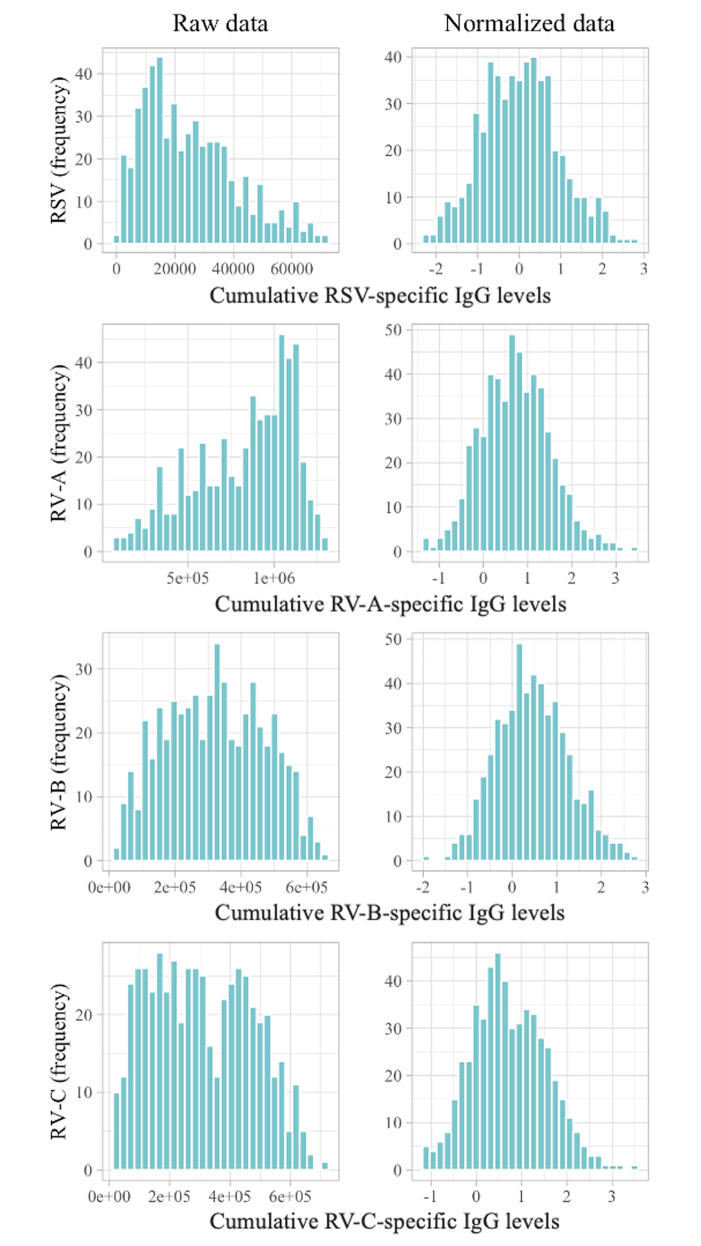
**SI Figure E2: Distribution of RV-specific and RSV-specific IgG levels before and after the normalization, in children (part A) and in adults (part B).** The ordered quantile normalization was applied to the whole population composed of both children and adults.

**Supplementary Figure E2B:**
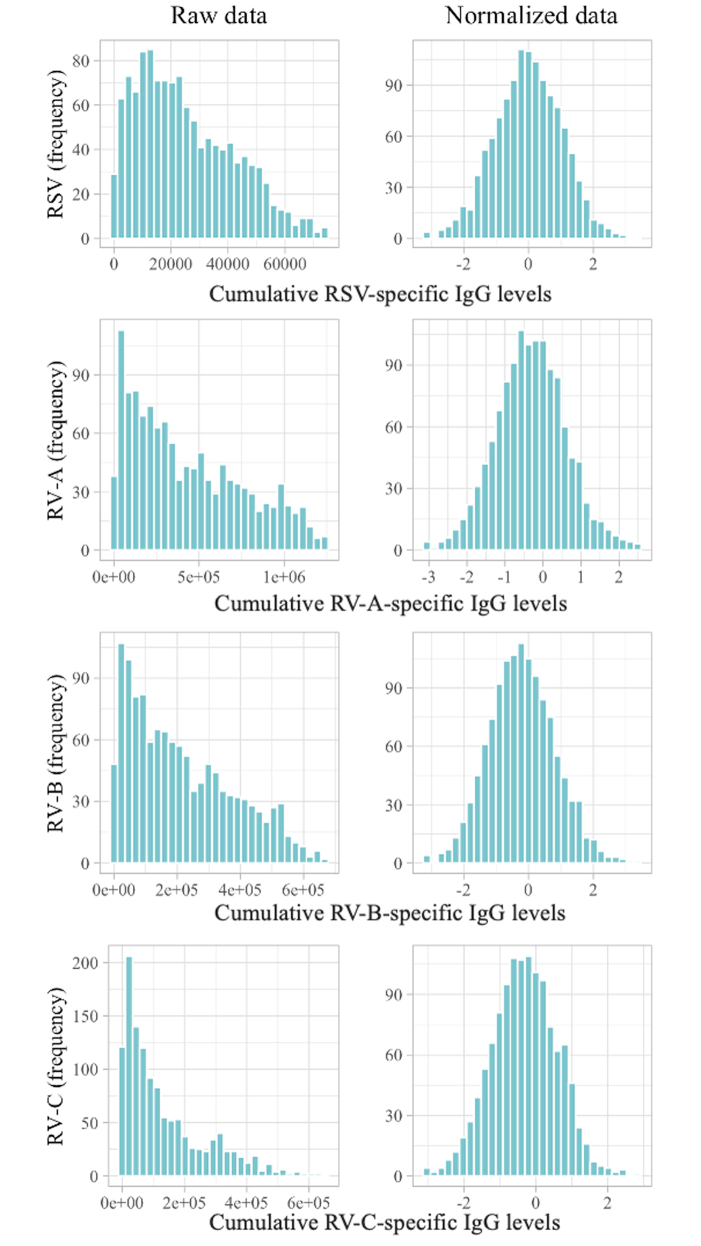
**SI Figure E2: Distribution of RV-specific and RSV-specific IgG levels before and after the normalization, in children (part A) and in adults (part B).** The ordered quantile normalization was applied to the whole population composed of both children and adults.
