# Supplementary material for: Association between asthma and IgG levels specific for rhinovirus and respiratory syncytial virus antigens in children and adults
Source: J Allergy Clin Immunol Glob. 2024 Sep 17;4(1):100342. doi: 10.1016/j.jacig.2024.100342 (PMC11536052; doi:10.1016/j.jacig.2024.100342)
Supplement: Supplementary Material [file mmc1.docx]

**Online supplementals**

**Methods**

**Inclusion criteria in the population**

The inclusion criteria for probands were age (7 to 65 years old), place of birth (their parents should have been born in France), area of residence, family structure (either adult subject with a spouse and at least one child, or children with at least one sibling and two parents) and for subjects, a positive answer to each of the four following standardized questions: “*Have you ever had attacks of breathlessness at rest with wheezing?*” “*Have you ever had asthma attacks?*” “*Was this diagnosis confirmed by a physician?*” and “*Have you had an asthma attack in the last 12 months*?”

**Measurement of RSV-specific and RV-specific IgG ; From an article in press (*Guillien et al. 2024,* doi : 10.3389/fimmu.**[**2024.1355214**](callto:2024.1355214)**)**

For the determination of virus-specific IgG antibodies, microarrays were processed as previously described (Niespodziana *et al.,* 2018, Niespodziana *et al*., 2021). Serum samples were diluted in two steps using an ImmunoCAP® Specific IgA/IgG Sample Diluent (Phadia, Uppsala, Sweden) to a final dilution of 1:300. Twenty slides per analysis run, each containing six arrays, were washed with phosphate-buffered saline with 0.1% Tween 20 (PBS/Tween) and dried by centrifugation using a Sigma 2–7 centrifuge and MTP-11113 rotor (both Sigma Laborzentrifugen GmbH, Osterode am Harz, Germany) for 2 min at 159 x g Thirty µl of 1:300 diluted sera, a Calibrator and sample diluent were applied onto each microarray and incubated for 2 hours at gentle rocking (6 times per minute) at room temperature (RT) (Biometra, Jena, Germany). Afterwards slides were washed again with PBS/Tween, dried by centrifugation and incubated with 30 µl/per array of the DyLight 550 (Pierce, Rockford, IL, USA) labelled anti-human IgG (1.8 µg/mL) (Jackson ImmunoResearch Laboratories, West Grove, PA, USA) for 30 min at RT. After further rinsing, washing with PBS/Tween and drying by centrifugation as described above, microarrays were scanned with a confocal PowerScanner from Tecan Grödig, Austria, using 30% of gain (i.e., photomultiplier (PMT)) and 10% of laser power.

Scanned images were analysed using the Mappix software (Innopsys, Carbonne, France). Fluorescence intensities (FI) of three replicated spots (i.e., raw data) were exported to an Excel file (Microsoft Corporation, Redmond, Washington, USA) and the median value of triplicate measurements was calculated.

**Standardization of RSV-specific and RV-specific IgG (From an article in press, Guillien et al. 2024, doi : 10.3389/fimmu.[2024.1355214](callto:2024.1355214))**

For the calibration and the determination of background signals, one Calibrator containing IgG antibodies against each of virus-derived antigens and one Sample Diluent (SD) (negative control), respectively, were included in each analysis run. During one analysis run approximately 120 arrays (one per serum sample) were analysed including the peptides in Table E1 The reproducibility of the assays was determined, by the coefficient of variation and intraclass correlation coefficient using sera from four individuals from EGEA2 by repeating measurements six times.

The specific raw-IgG data from 21 assay runs were first calibrated using the calibrator and negative control. Within each assay run, the difference between median value of the calibrator and median value of the sample diluent (SD) was calculated and the average of the differences was calculated for all 21 assay runs. Then we analyzed for each assay run the linear regression of this difference as a function of the average of the differences, with the peptide as the unit of study. Within each assay run, the calibrated value for a given peptide in a given participant corresponded to the median IgG value determined for the triplicates of each peptide divided by the linear regression slope. The background signals of all sample diluent repetitive measurements (n = 21) were calculated as a mean FI values + 3 SD for each antigen, and were subtracted from the measured FI reactivity values for each antigen. Values below the background level (specific to each IgG variables) were fixed to zero.

**Construction and normalization of the summary variables**

Summary variables for each RV species were calculated as the sum of the calibrated and corrected specific-IgG variables within each RV species. Summary variable for RSV was calculated as the calibrated and corrected raw RSV IgG variable.

We applied the Ordered Quantile Normalization transformation to the summary variables, using the “bestNormalize” R package, to obtain values within normal distribution.

**Statistical analysis and interpretation of statistical tests**

In agreement with the guidelines for Greenland and co-authors, interpretation of statistical tests was based on examining ORs magnitude and their 95%CI and precise P values (not whether P values are above or below 0.05) (25,26). Although the number of associations tested was relatively high (N=16) we did not apply any formal correction for multiple comparisons because 1) the study was based on a strong a priori hypothesis, 2) the 4 outcomes were highly interrelated and 3) the associations with asthma severity, age of asthma onset and the asthma symptoms score were aimed to dig the associations reported with the primary outcome “asthma ever” and thus should not be considered as independent tests.

**Interpretation of the asthma symptoms score and Mean Score Ratio (MSR)**

The asthma symptoms score has been defined by Pekkanen et al (22), and is considered as a count variable (categorial ordinal variable), suggesting a Poisson regression model. However, because of overdispersion, it requires the use of a negative binomial model.

The score was analyzed using a negative binomial model, which models the ratio of the mean score among exposed and nonexposed individuals. For example, a MSR of 1.63 for IgG levels means that a one-unit increase in IgG levels is associated with 63% higher mean score.

**Results**

**SI Figure E1: Beeswarm plots of RSV- and RV-specific antibody levels, in children (part A, EGEA1, n=530), and in adults (part B, EGEA2, n=1241).**

Distributions of virus-specific IgG data presented as median and interquartile range of the calibrated and corrected for the background level values (before normalization) and expressed in FI.

**SI Figure E2: Distribution of RV-specific and RSV-specific IgG levels before and after the normalization, in children (part A) and in adults (part B).**

The ordered quantile normalization was applied to the whole population composed of both children and adults.
