# Supplementary material for: Association between asthma and IgG levels specific for rhinovirus and respiratory syncytial virus antigens in children and adults
Source: J Allergy Clin Immunol Glob. 2024 Sep 17;4(1):100342. doi: 10.1016/j.jacig.2024.100342 (PMC11536052; doi:10.1016/j.jacig.2024.100342)
Supplement: Supplementary Tables [file mmc2.docx]

**SI Table E1: Distribution of RSV, RV-A, RV-B and RV-C according to the asthma ever status, in children (in EGEA1) and adults (in EGEA2)**

Distributions of virus-specific IgG data are presented as median [interquartile range] of the calibrated and corrected for the background level values (before normalization) and expressed in FI. For each RV species, the sum of the n peptides corresponds to the cumulative RV-specific IgG levels, and the mean over n peptides corresponds to the average antibody levels specific to RV-A, RV-B, RV-C.

|  |  | In children (n=530) | | In adults (n=1241) | |
| --- | --- | --- | --- | --- | --- |
|  |  |  |  |  |  |
|  |  | Asthmatics (n=274) | Non asthmatics (n=256) | Asthmatics (n=498) | Non asthmatics (n=743) |
| RSV | Specific IgG level  (1 antigen) | 24139  [12957-36224] | 19889  [11490-33675] | 20829  [9734-37865] | 22475  [12676-36738] |
| RV-A | Sum of the 18 peptides, median(IQR) | 918493  [704236-1070947] | 848379  [551257-1048015] | 352390  [137448-698541] | 343269  [158879-658835] |
|  | Mean over the 18 peptides, median(IQR) | 51027  [39124-59497] | 47132  [30625-58223] | 19577  [7636-38808] | 19070  [8827-36602] |
| RV-B | Sum of the 9 peptides, median(IQR) | 325754  [206779-432295] | 312658  [195077-440749] | 171559  [58015-324169] | 183902  [81194-329566] |
|  | Mean over the 9 peptides, median(IQR) | 36194  [22975-48032] | 34740  [21675-48972] | 19062  [6446-36019] | 20434  [9022-36618] |
| RV-C | Sum of the 10 peptides, median(IQR) | 309142  [183815-451179] | 253380  [145071-420565] | 80496  [28266-216681] | 87844  [34679-187602] |
|  | Mean over the 10 peptides, median(IQR) | 30914  [18381-45118] | 25338  [14507-42056] | 8050  [2827-21668] | 8784  [3468-18760] |

**SI Table E2: Association-study of RV-A, RV-B, and RV-C with asthma related outcomes stratified regarding allergic sensitization**

Multivariate associations between asthma related outcomes and RSV and RV-specific IgG levels in children and adults, among the subgroup with allergic sensitization and the subgroup without allergic sensitization, in children and adults.

Allergic sensitization was defined by a positive result to at least one of the 11 (in EGEA1) or 12 (in EGEA2) allergen extract by skin prick test.

Models were adjusted in EGEA1 on age, sex, BMI, season of blood sample and allergic sensitization and further adjusted at EGEA2 on tobacco active smoking.

In these analyses, models do not account for random effect on family, in order to preserve the convergence of the models.

* Estimates for asthma symptom score are MSR (Mean Score Ratio) which model the ratio of the mean asthma symptom score for each one-unit increase in virus IgG responses.

EGEA: Epidemiological study on the Genetics and Environment of Asthma; RSV: respiratory syncytial virus; RV: rhinovirus; CI: confidence interval; BMI: body mass index.

|  | **EGEA1 (n asthmatics = 269/520)** | | | | | |  | | **EGEA2 (n asthmatics = 470/1174)** | | | | | |
| --- | --- | --- | --- | --- | --- | --- | --- | --- | --- | --- | --- | --- | --- | --- |
|  | **No allergic sensitization**  **(n = 189)** | |  | **Allergic sensitization**  **( n = 331)** | | |  | | **No allergic sensitization**  **( n = 526)** | | | **Allergic sensitization**  **( n = 648)** | |  |
|  | **Adjusted OR [95%CI]** | **p** |  | **Adjusted OR [95%CI]** | **p** | **p inter**  **action** | | **Adjusted OR [95%CI]** | | **p** |  | **Adjusted OR [95%CI]** | **p** | **p inter**  **action** |
| Ever asthma |  |  |  |  |  |  | |  |  |  |  |  |  |  |
| RSV | 0.95 [0.65 ; 1.39] | 0.80 |  | 1.53 [1.14 ; 2.04] | 4.49e-03 | 0.08 | |  | 0.96 [0.75 ; 1.22] | 0.73 |  | 1.02 [0.88 ; 1.19] | 0.79 | 0.88 |
| RV-A | 1.39 [0.86 ; 2.26] | 0.18 |  | 1.68 [1.18 ; 2.38] | 3.78e-03 | 0.66 | |  | 0.84 [0.64 ; 1.10] | 0.21 |  | 0.85 [0.71 ; 1.03] | 0.09 | 0.55 |
| RV-B | 0.87 [0.55 ; 1.38] | 0.55 |  | 1.02 [0.73 ; 1.44] | 0.89 | 0.46 | |  | 0.84 [0.65 ; 1.07] | 0.15 |  | 0.83 [0.71 ; 0.98] | 0.03 | 0.74 |
| RV-C | 1.26 [0.79 ; 1.99] | 0.33 |  | 1.60 [1.16 ; 2.22] | 4.38e-03 | 0.65 | |  | 0.87 [0.66 ; 1.15] | 0.32 |  | 0.83 [0.69 ; 1.00] | 0.05 | 0.73 |
| Asthma severity |  |  |  |  |  |  | |  |  |  |  |  |  |  |
| RSV |  |  |  |  |  |  | |  |  |  |  |  |  |  |
| No asthma | 1.00 |  |  | 1.00 |  |  | |  | 1.00 |  |  | 1.00 |  |  |
| Mild asthma | 0.65 [0.38 ; 1.10] | 0.10 |  | 1.51 [1.10 ; 2.06] | 0.01 | 0.02 | |  | 0.97 [0.73 ; 1.29] | 0.83 |  | 1.03 [0.87 ; 1.21] | 0.73 | 0.87 |
| Moderate-to-severe asthma | 1.36 [0.68 ; 2.75] | 0.39 |  | 1.68 [1.12 ; 2.51] | 0.01 | 0.38 | |  | 0.86 [0.47 ; 1.56] | 0.61 |  | 0.92 [0.70 ; 1.21] | 0.54 | 0.99 |
| RV-A |  |  |  |  |  |  | |  |  |  |  |  |  |  |
| No asthma | 1.00 |  |  | 1.00 |  |  | |  | 1.00 |  |  | 1.00 |  |  |
| Mild asthma | 1.08 [0.56 ; 2.08] | 0.82 |  | 1.67 [1.15 ; 2.43] | 7.83e-03 | 0.40 | |  | 0.76 [0.55 ; 1.05] | 0.09 |  | 0.88 [0.72 ; 1.07] | 0.19 | 0.28 |
| Moderate-to-severe asthma | 1.18 [0.47 ; 2.93] | 0.72 |  | 1.84 [1.14 ; 2.96] | 0.01 | 0.54 | |  | 0.70 [0.34 ; 1.44] | 0.33 |  | 0.73 [0.53 ; 1.02] | 0.07 | 0.39 |
| RV-B |  |  |  |  |  |  | |  |  |  |  |  |  |  |
| No asthma | 1.00 |  |  | 1.00 |  |  | |  | 1.00 |  |  | 1.00 |  |  |
| Mild asthma | 0.79 [0.42 ; 1.48] | 0.46 |  | 1.02 [0.71 ; 1.47] | 0.91 | 0.30 | |  | 0.82 [0.61 ; 1.09] | 0.17 |  | 0.84 [0.71 ; 1.00] | 0.05 | 0.62 |
| Moderate-to-severe asthma | 0.50 [0.21 ; 1.23] | 0.13 |  | 1.01 [0.63 ; 1.60] | 0.97 | 0.26 | |  | 0.58 [0.30 ; 1.13] | 0.11 |  | 0.71 [0.53 ; 0.96] | 0.03 | 0.27 |
| RV-C |  |  |  |  |  |  | |  |  |  |  |  |  |  |
| No asthma | 1.00 |  |  | 1.00 |  |  | |  | 1.00 |  |  | 1.00 |  |  |
| Mild asthma | 1.12 [0.6 ; 2.11] | 0.72 |  | 1.44 [1.01 ; 2.05] | 0.04 | 0.67 | |  | 0.82 [0.59 ; 1.13] | 0.22 |  | 0.85 [0.70 ; 1.04] | 0.11 | 0.50 |
| Moderate-to-severe asthma | 1.12 [0.48 ; 2.64] | 0.79 |  | 2.21 [1.40 ; 3.47] | 6.99e-04 | 0.30 | |  | 0.73 [0.35 ; 1.49] | 0.39 |  | 0.76 [0.55 ; 1.06] | 0.11 | 0.42 |
| Age of asthma onset |  |  |  |  |  |  | |  |  |  |  |  |  |  |
| RSV |  |  |  |  |  |  | |  |  |  |  |  |  |  |
| No asthma | 1.00 |  |  | 1.00 |  |  | |  | 1.00 |  |  | 1.00 |  |  |
| ≤4 years | 0.94 [0.56 ; 1.58] | 0.82 |  | 1.59 [1.15 ; 2.19] | 4.86e-03 | 0.26 | |  | 0.48 [0.23 ; 0.98] | 0.04 |  | 1.22 [0.98 ; 1.52] | 0.07 | 0.04 |
| ]4-16] years | 0.92 [0.52 ; 1.64] | 0.78 |  | 1.43 [1.01 ; 2.02] | 0.04 | 0.08 | |  | 1.43 [0.89 ; 2.29] | 0.14 |  | 0.92 [0.75 ; 1.13] | 0.42 | 0.05 |
| >16 years |  |  |  |  |  |  | |  | 0.96 [0.71 ; 1.31] | 0.81 |  | 0.89 [0.70 ; 1.13] | 0.35 | 0.93 |
| RV-A |  |  |  |  |  |  | |  |  |  |  |  |  |  |
| No asthma | 1.00 |  |  | 1.00 |  |  | |  | 1.00 |  |  | 1.00 |  |  |
| ≤4 years | 1.38 [0.68 ; 2.79] | 0.37 |  | 1.67 [1.13 ; 2.45] | 9.79e-03 | 0.85 | |  | 0.43 [0.19 ; 0.99] | 0.05 |  | 0.86 [0.67 ; 1.10] | 0.23 | 0.14 |
| ]4-16] years | 1.24 [0.62 ; 2.49] | 0.54 |  | 1.79 [1.18 ; 2.70] | 6.06e-03 | 0.26 | |  | 1.40 [0.84 ; 2.32] | 0.19 |  | 0.80 [0.63 ; 1.02] | 0.07 | 0.03 |
| >16 years |  |  |  |  |  |  | |  | 0.75 [0.53 ; 1.07] | 0.12 |  | 0.83 [0.61 ; 1.13] | 0.24 | 0.93 |
| RV-B |  |  |  |  |  |  | |  |  |  |  |  |  |  |
| No asthma | 1.00 |  |  | 1.00 |  |  | |  | 1.00 |  |  | 1.00 |  |  |
| ≤4 years | 0.97 [0.50 ; 1.89] | 0.93 |  | 1.28 [0.88 ; 1.87] | 0.19 | 0.47 | |  | 0.63 [0.31 ; 1.28] | 0.20 |  | 0.86 [0.69 ; 1.08] | 0.20 | 0.41 |
| ]4-16] years | 0.64 [0.33 ; 1.25] | 0.19 |  | 0.78 [0.52 ; 1.17] | 0.23 | 0.52 | |  | 1.40 [0.87 ; 2.24] | 0.17 |  | 0.77 [0.62 ; 0.96] | 0.02 | 0.02 |
| >16 years |  |  |  |  |  |  | |  | 0.73 [0.53 ; 1.00] | 0.05 |  | 0.78 [0.60 ; 1.02] | 0.07 | 0.96 |
| RV-C |  |  |  |  |  |  | |  |  |  |  |  |  |  |
| No asthma | 1.00 |  |  | 1.00 |  |  | |  | 1.00 |  |  | 1.00 |  |  |
| ≤4 years | 1.31 [0.65 ; 2.65] | 0.45 |  | 1.45 [1.01 ; 2.08] | 0.04 | 0.75 | |  | 0.7 [0.31 ; 1.58] | 0.39 |  | 0.79 [0.61 ; 1.02] | 0.07 | 0.57 |
| ]4-16] years | 1.14 [0.60 ; 2.16] | 0.68 |  | 1.90 [1.29 ; 2.80] | 1.32e-03 | 0.19 | |  | 1.10 [0.65 ; 1.87] | 0.73 |  | 0.86 [0.67 ; 1.09] | 0.21 | 0.28 |
| >16 years |  |  |  |  |  |  | |  | 0.82 [0.57 ; 1.16] | 0.26 |  | 0.76 [0.56 ; 1.04] | 0.08 | 0.57 |
| Asthma symptoms score* |  |  |  |  |  |  | |  |  |  |  |  |  |  |
| RSV | 1.05 [0.76 ; 1.44] | 0.77 |  | 1.16 [1.03 ; 1.31] | 0.01 | 0.08 | |  | 1.02 [0.89 ; 1.17] | 0.73 |  | 0.98 [0.90 ; 1.06] | 0.57 | 0.88 |
| RV-A | 1.10 [0.73 ; 1.66] | 0.64 |  | 1.16 [1.01 ; 1.33] | 0.04 | 0.66 | |  | 0.98 [0.84 ; 1.14] | 0.76 |  | 0.90 [0.82 ; 0.99] | 0.03 | 0.55 |
| RV-B | 0.70 [0.48 ; 1.02] | 0.06 |  | 1 [0.87 ; 1.14] | 0.97 | 0.46 | |  | 0.98 [0.86 ; 1.13] | 0.79 |  | 0.90 [0.83 ; 0.97] | 8.79e-03 | 0.74 |
| RV-C | 1.13 [0.76 ; 1.66] | 0.55 |  | 1.17 [1.02 ; 1.33] | 0.02 | 0.65 | |  | 1.03 [0.88 ; 1.21] | 0.68 |  | 0.92 [0.84 ; 1.01] | 0.08 | 0.73 |
| Asthma associated with ICS use in the last 12 monts |  |  |  |  |  |  | |  |  |  |  |  |  |  |
| RSV |  |  |  |  |  |  | |  |  |  |  |  |  |  |
| No asthma | 1.00 |  |  | 1.00 |  |  | |  | 1.00 |  |  | 1.00 |  |  |
| Ever asthma without ICS | 0.76 [0.38 ; 1.55] | 0.46 |  | 1.27 [0.76 ; 2.12] | 0.36 | 0.24 | |  | 0.81 [0.58 ; 1.12] | 0.19 |  | 1.06 [0.89 ; 1.25] | 0.54 | 0.29 |
| Ever asthma with ICS | 1.14 [0.7 ; 1.86] | 0.59 |  | 1.6 [1.18 ; 2.17] | 2.38e-03 | 0.20 | |  | 1.15 [0.82 ; 1.62] | 0.41 |  | 0.97 [0.8 ; 1.18] | 0.77 | 0.32 |
| RV-A |  |  |  |  |  |  | |  |  |  |  |  |  |  |
| No asthma | 1.00 |  |  | 1.00 |  |  | |  | 1.00 |  |  | 1.00 |  |  |
| Ever asthma without ICS | 1.18 [0.52 ; 2.67] | 0.7 |  | 1.66 [0.92 ; 3] | 0.09 | 0.49 | |  | 0.85 [0.59 ; 1.21] | 0.35 |  | 0.95 [0.77 ; 1.17] | 0.6 | 0.69 |
| Ever asthma with ICS | 1.7 [0.92 ; 3.12] | 0.09 |  | 1.72 [1.2 ; 2.47] | 3.09e-03 | 0.99 | |  | 0.83 [0.56 ; 1.22] | 0.33 |  | 0.72 [0.57 ; 0.91] | 5.99e-03 | 0.74 |
| RV-B |  |  |  |  |  |  | |  |  |  |  |  |  |  |
| No asthma | 1.00 |  |  | 1.00 |  |  | |  | 1.00 |  |  | 1.00 |  |  |
| Ever asthma without ICS | 0.87 [0.39 ; 1.96] | 0.74 |  | 1.09 [0.6 ; 1.99] | 0.77 | 0.51 | |  | 0.77 [0.56 ; 1.08] | 0.13 |  | 0.85 [0.71 ; 1.02] | 0.09 | 0.71 |
| Ever asthma with ICS | 0.78 [0.44 ; 1.38] | 0.4 |  | 1.08 [0.76 ; 1.53] | 0.67 | 0.56 | |  | 0.93 [0.67 ; 1.31] | 0.68 |  | 0.79 [0.64 ; 0.97] | 0.02 | 0.98 |
| RV-C |  |  |  |  |  |  | |  |  |  |  |  |  |  |
| No asthma | 1.00 |  |  | 1.00 |  |  | |  | 1.00 |  |  | 1.00 |  |  |
| Ever asthma without ICS | 0.91 [0.42 ; 1.97] | 0.82 |  | 1.45 [0.84 ; 2.51] | 0.18 | 0.42 | |  | 0.83 [0.58 ; 1.2] | 0.32 |  | 0.93 [0.75 ; 1.15] | 0.48 | 0.58 |
| Ever asthma with ICS | 1.7 [0.96 ; 2.99] | 0.07 |  | 1.6 [1.15 ; 2.23] | 5.53e-03 | 0.89 | |  | 0.9 [0.61 ; 1.33] | 0.59 |  | 0.7 [0.56 ; 0.89] | 2.95e-03 | 0.85 |

**SI Table E3. Sensitivity analysis: association-study of RV-A-, RV-B-, RV-C-, RSV-specific IgG levels with asthma phenotypes stratified by the allergic sensitization status, conducted on a restricted population including a single member per family, in children (EGEA1) and in adults (EGEA2).**

This analysis was performed in a restricted population including only one member of each family, selected randomly, to address the robustness of the results to the study design.

The model is adjusted on age, sex, BMI, season of blood sample in EGEA1, and the same set of variables plus tobacco active smoking in EGEA2.

* Results are presented as Mean Score Ratio (MSR) and [95% CI]

|  | **EGEA1 (n asthmatics = 149/321)** | | | | | |  | **EGEA2 (n asthmatics = 166/489)** | | | | | |
| --- | --- | --- | --- | --- | --- | --- | --- | --- | --- | --- | --- | --- | --- |
|  | **No allergic sensitization**  **(n = 127)** | |  | **Allergic sensitization (n = 194)** | | |  | **No allergic sensitization**  **(n = 256)** | |  | **Allergic sensitization**  **(n = 233)** | |  |
|  | **Adjusted OR**  **[95%CI]** | **p** |  | **Adjusted OR [95%CI]** | **p** | **p inter**  **action** | | **Adjusted OR [95%CI]** | **p** |  | **Adjusted OR [95%CI]** | **p** | **p inter**  **action** |
| Ever asthma |  |  |  |  |  |  |  |  |  |  |  |  |  |
| RSV | 1.13 [0.69 ; 1.85] | 0.63 |  | 1.77 [1.21 ; 2.60] | 3.46e-03 | 0.06 |  | 1.07 [0.74 ; 1.56] | 0.71 |  | 1.05 [0.84 ; 1.33] | 0.66 | 0.88 |
| RV-A | 1.33 [0.71 ; 2.47] | 0.37 |  | 2.03 [1.30 ; 3.15] | 1.87e-03 | 0.14 |  | 0.88 [0.57 ; 1.34] | 0.54 |  | 0.84 [0.63 ; 1.10] | 0.20 | 0.83 |
| RV-B | 1.12 [0.63 ; 1.99] | 0.69 |  | 1.12 [0.73 ; 1.71] | 0.60 | 0.53 |  | 0.85 [0.58 ; 1.27] | 0.43 |  | 0.84 [0.65 ; 1.07] | 0.16 | 0.90 |
| RV-C | 1.18 [0.66 ; 2.12] | 0.57 |  | 2.03 [1.31 ; 3.13] | 1.57e-03 | 0.18 |  | 0.92 [0.60 ; 1.42] | 0.71 |  | 0.81 [0.61 ; 1.08] | 0.15 | 0.62 |
| Asthma severity |  |  |  |  |  |  |  |  |  |  |  |  |  |
| RSV |  |  |  |  |  |  |  |  |  |  |  |  |  |
| No asthma | 1.00 |  |  | 1.00 |  |  |  | 1.00 |  |  | 1.00 |  |  |
| Mild asthma | 0.67 [0.35 ; 1.30] | 0.24 |  | 1.87 [1.24 ; 2.83] | 3.31e-03 | 0.01 |  | 0.99 [0.64 ; 1.53] | 0.96 |  | 1.16 [0.91 ; 1.49] | 0.23 | 0.68 |
| Moderate-to-severe asthma | 1.56 [0.64 ; 3.80] | 0.33 |  | 1.83 [1.06 ; 3.15] | 0.03 | 0.30 |  | 3.89 [0.64 ; 23.55] | 0.14 |  | 0.53 [0.32 ; 0.89] | 0.02 | 0.11 |
| RV-A |  |  |  |  |  |  |  |  |  |  |  |  |  |
| No asthma | 1.00 |  |  | 1.00 |  |  |  | 1.00 |  |  | 1.00 |  |  |
| Mild asthma | 0.95 [0.41 ; 2.15] | 0.89 |  | 1.83 [1.14 ; 2.95] | 0.01 | 0.11 |  | 0.73 [0.44 ; 1.19] | 0.20 |  | 0.89 [0.66 ; 1.19] | 0.42 | 0.64 |
| Moderate-to-severe asthma | 1.04 [0.34 ; 3.16] | 0.95 |  | 2.06 [1.10 ; 3.85] | 0.02 | 0.38 |  | 2.83 [0.37 ; 21.70] | 0.31 |  | 0.50 [0.28 ; 0.89] | 0.02 | 0.41 |
| RV-B |  |  |  |  |  |  |  |  |  |  |  |  |  |
| No asthma | 1.00 |  |  | 1.00 |  |  |  | 1.00 |  |  | 1.00 |  |  |
| Mild asthma | 0.97 [0.46 ; 2.07] | 0.94 |  | 1.17 [0.73 ; 1.88] | 0.50 | 0.26 |  | 0.75 [0.47 ; 1.19] | 0.22 |  | 0.87 [0.66 ; 1.14] | 0.3 | 0.62 |
| Moderate-to-severe asthma | 0.54 [0.19 ; 1.58] | 0.26 |  | 0.81 [0.42 ; 1.54] | 0.51 | 0.54 |  | 1.39 [0.33 ; 5.82] | 0.65 |  | 0.47 [0.26 ; 0.83] | 9.22e-03 | 0.38 |
| RV-C |  |  |  |  |  |  |  |  |  |  |  |  |  |
| No asthma | 1.00 |  |  | 1.00 |  |  |  | 1.00 |  |  | 1.00 |  |  |
| Mild asthma | 1.04 [0.48 ; 2.26] | 0.93 |  | 1.66 [1.04 ; 2.64] | 0.03 | 0.27 |  | 0.80 [0.48 ; 1.33] | 0.38 |  | 0.88 [0.65 ; 1.19] | 0.41 | 0.84 |
| Moderate-to-severe asthma | 1.24 [0.43 ; 3.52] | 0.69 |  | 2.63 [1.42 ; 4.89] | 2.38e-03 | 0.37 |  | 8.44 [0.20 ; 348.04] | 0.26 |  | 0.54 [0.30 ; 0.98] | 0.04 | 0.32 |
| Age of asthma onset |  |  |  |  |  |  |  |  |  |  |  |  |  |
| RSV |  |  |  |  |  |  |  |  |  |  |  |  |  |
| No asthma | 1.00 |  |  | 1.00 |  |  |  | 1.00 |  |  | 1.00 |  |  |
| ≤4 years | 1.06 [0.58 ; 1.97] | 0.84 |  | 1.9 [1.23 ; 2.92] | 3.97e-03 | 0.15 |  | 0.17 [0.04 ; 0.84] | 0.03 |  | 1.47 [1.02 ; 2.12] | 0.04 | 0.01 |
| ]4-16] years | 0.93 [0.43 ; 1.98] | 0.84 |  | 1.6 [1.00 ; 2.56] | 0.05 | 0.04 |  | 1.49 [0.70 ; 3.19] | 0.30 |  | 0.79 [0.57 ; 1.09] | 0.15 | 0.11 |
| >16 years |  |  |  |  |  |  |  | 1.18 [0.73 ; 1.89] | 0.50 |  | 0.97 [0.66 ; 1.42] | 0.87 | 0.73 |
| RV-A |  |  |  |  |  |  |  |  |  |  |  |  |  |
| No asthma | 1.00 |  |  | 1.00 |  |  |  | 1.00 |  |  | 1.00 |  |  |
| ≤4 years | 1.34 [0.56 ; 3.16] | 0.51 |  | 2.00 [1.21 ; 3.30] | 7.06e-03 | 0.5 |  | 0.08 [0.01 ; 0.77] | 0.03 |  | 1.00 [0.67 ; 1.50] | 1.00 | 0.04 |
| ]4-16] years | 1.02 [0.43 ; 2.43] | 0.97 |  | 2.08 [1.22 ; 3.57] | 7.83e-03 | 0.08 |  | 1.53 [0.70 ; 3.34] | 0.28 |  | 0.63 [0.44 ; 0.92] | 0.02 | 0.04 |
| >16 years |  |  |  |  |  |  |  | 0.85 [0.49 ; 1.48] | 0.56 |  | 0.88 [0.54 ; 1.44] | 0.61 | 0.72 |
| RV-B |  |  |  |  |  |  |  |  |  |  |  |  |  |
| No asthma | 1.00 |  |  | 1.00 |  |  |  | 1.00 |  |  | 1.00 |  |  |
| ≤4 years | 1.46 [0.67 ; 3.19] | 0.34 |  | 1.61 [0.98 ; 2.66] | 0.06 | 0.59 |  | 0.16 [0.03 ; 0.9] | 0.04 |  | 0.97 [0.66 ; 1.40] | 0.86 | 0.05 |
| ]4-16] years | 0.62 [0.26 ; 1.45] | 0.27 |  | 0.72 [0.41 ; 1.23] | 0.23 | 0.49 |  | 1.53 [0.71 ; 3.30] | 0.28 |  | 0.63 [0.44 ; 0.90] | 0.01 | 0.04 |
| >16 years |  |  |  |  |  |  |  | 0.67 [0.39 ; 1.13] | 0.13 |  | 0.81 [0.52 ; 1.27] | 0.36 | 0.97 |
| RV-C |  |  |  |  |  |  |  |  |  |  |  |  |  |
| No asthma | 1.00 |  |  | 1.00 |  |  |  | 1.00 |  |  | 1.00 |  |  |
| ≤4 years | 1.19 [0.52 ; 2.69] | 0.68 |  | 1.85 [1.13 ; 3.02] | 0.01 | 0.64 |  | 0.21 [0.03 ; 1.36] | 0.10 |  | 0.83 [0.54 ; 1.26] | 0.38 | 0.23 |
| ]4-16] years | 0.92 [0.39 ; 2.13] | 0.84 |  | 2.31 [1.36 ; 3.92] | 2.24e-03 | 0.05 |  | 1.15 [0.51 ; 2.57] | 0.74 |  | 0.73 [0.50 ; 1.08] | 0.12 | 0.26 |
| >16 years |  |  |  |  |  |  |  | 0.95 [0.55 ; 1.66] | 0.86 |  | 0.87 [0.53 ; 1.44] | 0.59 | 0.48 |
| Asthma symptoms score* |  |  |  |  |  |  |  |  |  |  |  |  |  |
| RSV | 1.15 [0.81 ; 1.64] | 0.42 |  | 1.25 [1.05 ; 1.49] | 0.01 | 0.06 |  | 1.04 [0.84 ; 1.27] | 0.74 |  | 0.89 [0.78 ; 1.02] | 0.09 | 0.88 |
| RV-A | 1.05 [0.67 ; 1.63] | 0.84 |  | 1.29 [1.05 ; 1.58] | 0.02 | 0.14 |  | 1.11 [0.88 ; 1.41] | 0.38 |  | 0.83 [0.71 ; 0.96] | 0.02 | 0.83 |
| RV-B | 0.74 [0.49 ; 1.11] | 0.15 |  | 1.00 [0.81 ; 1.24] | 0.99 | 0.53 |  | 1.06 [0.85 ; 1.31] | 0.61 |  | 0.85 [0.74 ; 0.98] | 0.02 | 0.90 |
| RV-C | 1.21 [0.79 ; 1.86] | 0.38 |  | 1.30 [1.07 ; 1.58] | 9.79e-03 | 0.18 |  | 1.21 [0.95 ; 1.53] | 0.12 |  | 0.86 [0.73 ; 1.00] | 0.06 | 0.62 |
| Asthma associated with ICS use in the last 12 months |  |  |  |  |  |  |  |  |  |  |  |  |  |
| RSV |  |  |  |  |  |  |  |  |  |  |  |  |  |
| No asthma | 1.00 |  |  | 1.00 |  |  |  | 1.00 |  |  | 1.00 |  |  |
| Ever asthma without ICS | 0.98 [0.32 ; 2.96] | 0.97 |  | 1.35 [0.6 ; 3.03] | 0.46 | 0.51 |  | 0.87 [0.55 ; 1.39] | 0.57 |  | 1.08 [0.83 ; 1.41] | 0.57 | 0.68 |
| Ever asthma with ICS | 1.26 [0.69 ; 2.31] | 0.46 |  | 1.84 [1.24 ; 2.74] | 2.4e-03 | 0.07 |  | 1.51 [0.82 ; 2.77] | 0.19 |  | 1.02 [0.76 ; 1.39] | 0.88 | 0.33 |
| RVA |  |  |  |  |  |  |  |  |  |  |  |  |  |
| No asthma | 1.00 |  |  | 1.00 |  |  |  | 1.00 |  |  | 1.00 |  |  |
| Ever asthma without ICS | 0.77 [0.21 ; 2.84] | 0.69 |  | 2.35 [0.93 ; 5.97] | 0.07 | 0.16 |  | 0.81 [0.49 ; 1.35] | 0.42 |  | 0.85 [0.62 ; 1.17] | 0.32 | 0.84 |
| Ever asthma with ICS | 1.75 [0.82 ; 3.73] | 0.15 |  | 1.98 [1.27 ; 3.09] | 2.67e-03 | 0.39 |  | 1 [0.51 ; 1.97] | 1 |  | 0.8 [0.55 ; 1.14] | 0.21 | 0.99 |
| RVB |  |  |  |  |  |  |  |  |  |  |  |  |  |
| No asthma | 1.00 |  |  | 1.00 |  |  |  | 1.00 |  |  | 1.00 |  |  |
| Ever asthma without ICS | 1.64 [0.47 ; 5.76] | 0.44 |  | 1.48 [0.63 ; 3.51] | 0.37 | 0.77 |  | 0.85 [0.52 ; 1.39] | 0.52 |  | 0.81 [0.6 ; 1.08] | 0.16 | 0.71 |
| Ever asthma with ICS | 0.97 [0.5 ; 1.88] | 0.92 |  | 1.1 [0.72 ; 1.69] | 0.65 | 0.55 |  | 0.92 [0.5 ; 1.68] | 0.78 |  | 0.86 [0.62 ; 1.19] | 0.37 | 0.75 |
| RVC |  |  |  |  |  |  |  |  |  |  |  |  |  |
| No asthma | 1.00 |  |  | 1.00 |  |  |  | 1.00 |  |  | 1.00 |  |  |
| Ever asthma without ICS | 0.74 [0.23 ; 2.41] | 0.61 |  | 2.08 [0.85 ; 5.12] | 0.11 | 0.19 |  | 0.75 [0.44 ; 1.3] | 0.31 |  | 0.87 [0.63 ; 1.22] | 0.43 | 0.93 |
| Ever asthma with ICS | 1.66 [0.83 ; 3.3] | 0.15 |  | 1.99 [1.28 ; 3.1] | 2.2e-03 | 0.49 |  | 1.29 [0.65 ; 2.56] | 0.47 |  | 0.72 [0.49 ; 1.04] | 0.08 | 0.37 |
